# ADPredict: ADP-ribosylation site prediction based on physicochemical and structural descriptors

**DOI:** 10.1093/bioinformatics/bty159

**Published:** 2018-03-15

**Authors:** Matteo Lo Monte, Candida Manelfi, Marica Gemei, Daniela Corda, Andrea Rosario Beccari

**Affiliations:** 1 Institute of Protein Biochemistry, National Research Council, Naples, Italy; 2 Dompé Farmaceutici SpA, L'Aquila; 3 Dipartimento di Scienze Farmaceutiche, Università degli Studi di Milano, Milano, Italy

## Abstract

**Motivation:**

ADP-ribosylation is a post-translational modification (PTM) implicated in several crucial cellular processes, ranging from regulation of DNA repair and chromatin structure to cell metabolism and stress responses. To date, a complete understanding of ADP-ribosylation targets and their modification sites in different tissues and disease states is still lacking. Identification of ADP-ribosylation sites is required to discern the molecular mechanisms regulated by this modification. This motivated us to develop a computational tool for the prediction of ADP-ribosylated sites.

**Results:**

Here, we present *ADPredict*, the first dedicated computational tool for the prediction of ADP-ribosylated aspartic and glutamic acids. This predictive algorithm is based on (i) physicochemical properties, (ii) in-house designed secondary structure-related descriptors and (iii) three-dimensional features of a set of human ADP-ribosylated proteins that have been reported in the literature. *ADPredict* was developed using principal component analysis and machine learning techniques; its performance was evaluated both internally via intensive bootstrapping and in predicting two external experimental datasets. It outperformed the only other available ADP-ribosylation prediction tool, *ModPred*. Moreover, a novel secondary structure descriptor, *HM-ratio*, was introduced and successfully contributed to the model development, thus representing a promising tool for bioinformatics studies, such as PTM prediction.

**Availability and implementation:**

*ADPredict* is freely available at www.ADPredict.net.

**Supplementary information:**

Supplementary data are available at *Bioinformatics* online.

## 1 Introduction

Post-translational modifications (PTMs) exponentially increase the variety of protein functions in an organism, allowing fine-tuned and rapid responses to a wide range of stimuli occurring in both physiological and pathological conditions. PTMs can occur by covalent addition of functional groups or small molecules (such as phosphorylation or ubiquitination), as well as by redox modifications, bond formation or peptide cleavage (either degradative or activating). Due to improved detection technologies, the list of protein modifications in the literature has risen to well over 200 (Mann and Jensen, 2003; Olsen and Mann, 2013). Although some of these events have been exhaustively described, for many others, the actors involved, as well as the cellular environment and the cascade of downstream events, are only partially understood. This is the case for ADP-ribosylation biology, where although the understanding of its role in cell functions has been greatly enhanced in recent years, the related molecular mechanisms often remain to be explored since sites of modification have not been mapped in most cases (Gupte *et al.*, 2017).

ADP-ribosylation consists of the enzymatic transfer of the ADP-ribose (ADPr) moiety from nicotinamide adenine dinucleotide (NAD^+^) to a target protein, with release of nicotinamide (Ueda and Hayaishi, 1985). It is catalyzed by both ecto-enzymes [ecto-ADP-ribosyltransferases (ecto-ARTs)] and intracellular enzymes [poly-ADP-ribosylpolymerases (PARPs)] (Grimaldi *et al.*, 2015; Palazzo *et al.*, 2017; Ueda and Hayaishi, 1985). Ecto-ARTs specifically transfer a single unit of ADPr on arginine residues of membrane/extracellular targets (Laing *et al.*, 2011). Differently, enzymes of the PARP family can add either a single unit of ADPr (mono-ARTs) or multiple moieties to form long and branched ADPr polymers (PARPs), mostly on lysine and acidic residues (Messner *et al.*, 2010; Vyas *et al.*, 2014). Protein ADP-ribosylation is a heterogeneous, highly charged and rapidly degraded PTM. These features made difficult the identification of target residues for a long time and only in recent years, especially considering the low abundance of endogenous ADP-ribosylation at basal level (Larsen *et al.*, 2017).

In the last few years, the development of novel techniques to profile ADP-ribosylated proteins at the residue level has been reported (Bartolomei *et al.*, 2016; Bilan *et al.*, 2017a; Chapman *et al.*, 2013; Daniels *et al.*, 2014, 2017; Gibson *et al.*, 2016; Ismail *et al.*, 2015; Martello *et al.*, 2016; Zhang *et al.*, 2013). The increasing available information concerning both identified substrates and specific residues of the modification has been systematically collected (Vivelo *et al.*, 2017) and lays the groundwork for the development of ADP-ribosylation prediction tools. To date, two such tools have been published: ModPred, by Radivojac and colleagues (Pejaver *et al.*, 2014), it is not exclusively focused on ADP-ribosylation but rather meant as a multi-PTM predictor and ADPRtool, by Liu *et al.* (2015), which focuses on ADP-ribosylation of aspartic acid residues but is unfortunately not available to the community.

Thus, given the lack of a fully dedicated computational tool and taking advantage of the newest experimental data, we sought to design a specific algorithm to accurately predict ADP-ribosylation sites throughout the human proteome. Based on physicochemical properties and, when available, on structure-related information of a wide set of experimental ADP-ribosylated sites, ADPredict identifies the aspartic acid and glutamic acid most probable to be ADP-ribosylated within a target protein. We focused on these two residue types since the better-known ADP-ribosylation target and the most abundant among available experimental data.

Being able to provide robust and confident predictions, ADPredict would facilitate the biologist in investigating the molecular mechanisms underlying pathways of interest mediated by ADP-ribosylation.

## 2 Materials and methods

**Fig. 1.**
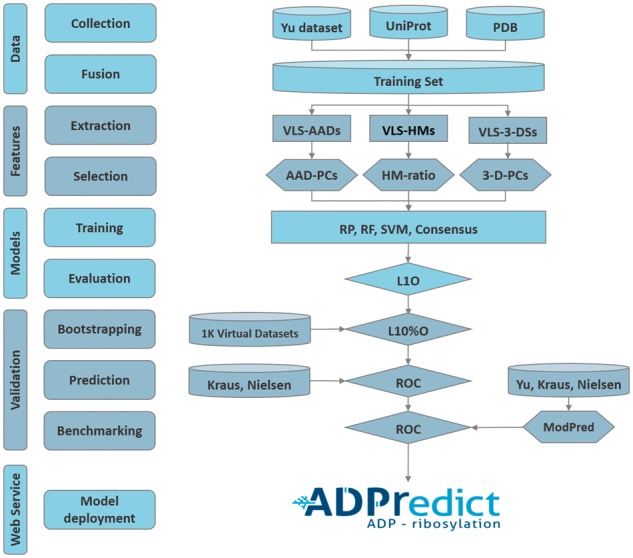
Schematic framework of ADPredict development. On the left, the vertical labels list the main stages of the study; relative itemized details follow. On the right, a diagram schematizes the activity flow

The overall framework is schematized in Figure 1, which shows the five main stages trough which ADPredict was developed. The first stage, data collection and data fusion, includes all pre-processing stages through which the training set was refined. The second stage, feature extraction and selection, comprises the calculations of all considered protein properties. Then, model training and evaluation refer to algorithm development and performance control and comparison. The fourth stage comprises 1000 runs of bootstrapping, the external prediction of two other datasets and a successful benchmarking session. The final step concerns web service deployment.

### 2.1 Data collection

#### 2.1.1 Pre-processing

The training dataset was generated starting from a collection of ADP-ribosylated sites identified via mass spectrometry published by Yu and colleagues (Zhang *et al.*, 2013). The set consists of a total of 1048 aspartic acid and glutamic acid residues belonging to 340 different human proteins. A data-cleaning session, aimed to standardize data entries, was performed. In details (i) when missing, primary sequence was retrieved from Uniprot databank and (ii) checked for congruence with the indicated international protein index or gene name; (iii) the position of modified residue within the primary sequence was controlled and corrected if the case; (iv) sub-sequences not included into the relative protein primary sequence (possibly due to incorrect isoform annotation) were excluded; (v) Titin protein (Q8WZ42), accounting for 6 modified sites and almost 5000 non modified glutamic and aspartic acids, was removed form dataset. This procedure led to a set of 1018 unique modified residues (821 glutamic acid and 197 aspartic acid) distributed across 317 proteins. For this set, protein sequences from the UniProt databank (The UniProt, 2017) and, when available, the related resolved structures from the PDB databank (Berman *et al.*, 2000) were retrieved. For this second aspect, a dataflow was specifically developed to properly select and retrieve only the most representative crystal structure. To do this, the related primary sequences were retrieved from the PDB and checked against the relative original primary sequences for mismatches or missing regions. Inconsistencies were corrected to properly perform the alignment. The criteria used to select the best structures were (i) maximum number of modified residues; (ii) minimum number of missing/erroneous residues; (iii) maximum portion of the protein resolved; and (iv) preference for a crystal structure, the most recently published, and with the best resolution possible. Several proteins showing only resolved structures not covering any modified site were discarded because they could not be used. In total, 54 protein structures featuring 135 ADP-ribosylated sites (91 glutamic acid and 44 aspartic acid) were collected.

#### 2.1.2 Descriptive analysis

**Fig. 2.**
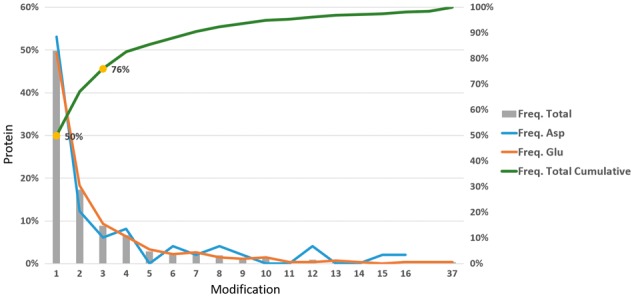
ADP-ribosylated site distribution among the training set proteins. Yellow dots mark the percentage of proteins reporting one or up to three modifications. Blue and orange lines refer to the count of modified aspartic acid and glutamic acid, respectively; gray bars display the percentage of proteins (left y-axis) with a certain number of modifications; and the green line represents the cumulative curve (right y-axis)

A detailed descriptive analysis was undertaken to exhaustively examine the data and to set up an appropriate computational strategy. The 1018 validated ADP-ribosylated sites were considered as true positives (TPs), while all other 29 757 acidic residues reported to be unmodified were considered as true negatives (TNs). The total number of TNs was ∼30 times larger than that of TPs. Since this unbalance would negatively influence our ability to develop a TP prediction algorithm, a selection of 5000 TNs (an arbitrarily chosen number) was made using the maximum dissimilarity method, as implemented in the Pipeline Pilot program (Warr, 2012), to preserve the representation of the entire physicochemical space of the dataset and to limit the introduced bias. Another crucial aspect for our final goal arises from the distribution of ADP-ribosylated sites among proteins in the set. Despite being twice longer than the expected, with a mean length of about 741 amino acids, the 317 proteins in the refined dataset showed a moderate rate of ADP-ribosylated sites. In details, for almost 50% of the proteins, only one TP was experimentally identified, increasing to more than 75% if we considered up to three modifications. This was true for both glutamic acid and aspartic acid residues (Fig. 2). On these grounds, models were tested and selected according to their capacity to correctly predict ADP-ribosylated sites within the top three positions, ranked according to the prediction score, of the acidic residues list of a target protein.

### 2.2 Feature extraction

Once the dataset had been refined and focused, and before proceeding with physicochemical- and structure-related feature extraction, the sub-sequences of interest were retrieved from the proteins’ full-length primary sequence, aiming to identify these features in the immediate vicinity of the modified site. A progressively wider window centered on the modified residue in the primary sequence was assessed to obtain sequence fragments of 5–33 amino acid residues [variable length sub-sequence (VLS)]. The lower limit was selected because a shorter fragment of three amino acids would not sufficiently describe a chemical or structural space, resulting in common triplets that are indistinguishable in terms of predictions. On the other hand, the upper limit was chosen based on structural evaluations, i.e. large enough to encompass a hypothetical large, meaningful folding motif, such as a membrane-spanning alpha helix or a beta sheet. Once the VLSs were extracted, both physicochemical- and structure-related scores were calculated. For the primary sequence-based features, we used a selection of the amino acid descriptor (AAD) sets reported in the literature, accounting for physicochemical as well as topological and three-dimensional electrostatic properties. Specifically, we annotated the first three principal components of Z-Scales, ST-Scales, Prot-FP and MSWHIM sets (Sandberg *et al.*, 1998; Yang *et al.*, 2010; Zaliani and Gancia, 1999), as previously reported and recently compared in terms of descriptive capacity by Bender and colleagues (van Westen *et al.*, 2013). A 12 score-per-amino-acid string was thus obtained (VLS-AAD), preserving the relative position of every single amino acid within the sub-sequence. Structure-related features were also taken into account, with the assumption that besides a specific chemistry, a proper shape is also necessary to allow the ADP-ribosyltransferase to properly approach the residue being modified. Thus, for those proteins whose structure has been published, folding was annotated as reported by the PDB databank dictionary. The classification based on seven motifs was translated into a simpler four-motif classification, with the aim to focus on the most significant structural classes (Supplementary Table S1). A helix-strand-turn-coil annotated sub-sequence list was therefore obtained, again with the variable length approach described before. These strings were further coded with an in-house-developed hashing code to identify only robust and meaningful folds, and if this was not the case, to add the uncertainty annotation (reported as 0). In detail, the hashing strategy consisted of splitting the simplified secondary structure string into three to seven fragments, depending on the VLS; within each fragment annotated folds are counted and the most numerous one is selected. In the case of two equally represented folds, an uncertainty is annotated (0). Therefore, three, five or seven-letter hashed strings were produced, which we refer to as VLS-hashed motifs (VLS-HMs) (Table 1). Moreover, the hashing code allowed us to reduce the number of diverse entries, especially for the very long VLSs and to make them comparable to each other, enabling their use in predictive algorithm development. As with motif folding, whole protein-based 3-D properties were calculated as well, in line with what has been done in other prediction tools (Brandes *et al.*, 2016) and based on the assumption that a modifiable residue would be chemically available to establish an interaction with the enzyme as well as exposed to the external environment. Therefore, 14 parameters accounting for structural information, such as solvent exposure (free and bound, namely FreeASA and GxG ASA, respectively), the number of rotatable bonds, the possible presence of intramolecular interactions and several intramolecular energies, were calculated with the molecular operating environment (MOE, 2018) and small-molecule drug discovery suite (Schrödinger, 2018) programs and reported for every amino acid, resulting in a 14 score-per-amino-acid string, called VLS-3-DSs (Supplementary Table S2).

### 2.3 Feature selection

*Principal component analysis* (PCA) was performed for both VLS-AADs and VLS-3-DScores, mean centered and scaled to unit variance and the principal components explaining 75% of the variance were used as descriptors for model development (AAD-PCs and 3-D-PCs, respectively). The frequency of TP and TN entries in VLS-HMs was calculated, and the resulting ratio was used as the structure-related descriptor (HM-ratio).

**Table 1.** Secondary structure hashing strategy

VLS	Primary structure	Secondary structure	Metrics	Hashed motif
9	MATTEWLMN	CHH–HHT–TTC	3-3-3	H–H–T
11	WMATTEWLMNT	CCH–HHHTT–TCE	3-5-3	C–H–0
13	YWMATTEWLMNTY	ECCH–HHHTT–TCEE	4-5-4	C–H–E
15	IYWMATTEWLMNTYA	EECCH–HHHTT–TCEEE	5-5-5	0–H–E

*Note*: Example of the hashing strategy exploited to annotate secondary structure information of the considered sub-sequences. The metrics accounts for the fragmentation of the annotated string. For each fragment, the most representative fold is taken; when not possible uncertainty is introduced (0).

## 3 Machine learning algorithms

### 3.1 Model training

Regression tree methods are very popular statistics tools that are used widely in many fields, including biological event exploration and prediction (Jia *et al.*, 2016a; Li *et al.*, 2015; Xiao *et al.*, 2016). Among these, we exploited the recursive partitioning (RP) and the random forest (RF) methods (Breiman, 1984, 2001) to interpret our dataset and to derive predictive models, as has been done previously (Jia *et al.*, 2016b; Xiao *et al.*, 2016). In addition to classification tree methods, we used as well a supervised learning technique statistical model, namely, the support vector machines model (SVM, also known as support vector network) (Cai and Jiang, 2016; Xu *et al.*, 2016). RP models were calculated with the R statistics module, Learn RP Tree model, embedded in Pipeline Pilot. The output consisted of differently pruned trees, from the most pruned one to the largest, completely unpruned one. The maximum tree depth was set to 50 as the number of maximum knots allowed per property; the Gini index was used for ranking the desirability of splits in the data. The internal ranking and selection of the best trees were made by evaluating the ROC function, which was automatically computed by the Pipeline Pilot component. Similarly, RF models were calculated with the R statistics module, Learn RP Forest model. Tree settings were set in line with those of RP models, and the bootstrap aggregation method was used in constructing the forest. Finally, the SVM models were developed using the R statistics module, Learn R SVM model, setting the kernel as radial and the C and ε parameters as 1.0 and 0.1, respectively. As for the regression tree methods, the output consisted of a possibility score for a site to be ADP-ribosylated, expressed as values between 0 and 1, with higher values reflecting higher probability. Three parallel model development campaigns were performed using AAD-PC, HM-ratio and 3-D-PC descriptors, separately, to initially obtain two different classes of models: one based on the primary sequence that would always be useable and a second developed from structure-related information and thus only exploitable when the structure of the protein of interest is known. These methods were applied to all VLSs, aiming to objectively identify the best performing sub-sequence length for our study.

### 3.2 Model evaluation

Enrichment factor (EF) (Efron and Tibshirani, 1993; Kirchmair *et al.*, 2008) and receiving operating characteristic (ROC) (Fawcett, 2006) were used as evaluation functions. The EF assessed the improvement of the hit rate of correctly predicted TPs compared to a random selection, considering the top three ranked residues (since more than 75% of the proteins in the dataset possess at most three ADP-ribosylated sites). When more residues than those considered as the top three were ranked within the selected positions of the prediction list due to an equal score, the sub-selection to calculate the models’ performance was extended to include all equally scored top ranked sites, which did not affect the results because the EFs were calculated considering the resulting number of involved residues. In this study, an EF threshold value for separating correctly and incorrectly predicted proteins was set at two (twice the random incidence). The ROC was selected to measure the global performance of the models (Shamsara, 2014), and the ROC curve was plotted to visualize the TP rate (sensitivity) against the false positive rates (1—specificity), calculated progressively while varying the TP/TN discriminating threshold from 0 to 1 in small increments. Models with higher EF values are preferred when global performances (ROC values) are not sufficient to discriminate them.

### 3.3 Cross-validation

Internal and external validations of the generated models and their statistical stability were evaluated. Cross-validation strategies, such as the leave-*n*-out, are widely used to overcome the intrinsic overfitting limits of machine learning. In this study, we performed both leave-one-out (L1O) and leave-10%-out (L10%O) calculations for performance evaluation and model stability assessment, respectively (Fu *et al.*, 2005; Kohavi, 1995). The models were re-generated each time after the removal of *n* proteins, and EF and ROC were calculated for each protein individually. The overall performance of the models was evaluated in terms of mean values of the evaluation functions and their standard deviations (SDEV-all). To assess the reproducibility of model performance, the standard deviations were also calculated as an average of all the proteins predicted for each run (SDEV-mean). As for the model development, cross-validation techniques were applied to all the VLSs separately, and the best performing model for each descriptor was identified. In particular, to accurately perform the L10%O, a two-step strategy for random number selection was designed and implemented. A selected set of 1000 lists comprising 32 proteins (10% of the dataset) each was chosen maximizing their diversity by the application of the fingerprint-based Maximum dissimilarity method. For the structure-related descriptors, the only difference lies in generating subsets of six proteins, since the structure was available for 54 proteins. The obtained lists were used to generate training and test sets for a 1000 runs bootstrap. This approach allowed us to avoid bias due to a non-random selection of the subsets for the bootstrapping stages (Efron and Tibshirani, 1993).

### 3.4 External validation

In addition to the work of Yu and colleagues, two studies about ADP-ribosylation site identification have been published more recently, both reporting a detailed list of experimentally derived ADP-ribosylated aspartic and glutamic acids. Once pre-processed—using the same protocol applied in refining the Yu dataset—the first study, published by Kraus and colleagues (Gibson *et al.*, 2016), resulted into 1150 modified sites, while the second one, published by Nielsen and colleagues (Martello *et al.*, 2016), accounted for 1137 modified sites (see Supplementary Fig. S2 for more details). We used these two external test sets to further evaluate the predictive capacity of the generated models, excluding from the calculation those modified proteins already present in the training set, to avoid the introduction any bias.

### 3.5 Benchmarking

The predictive performance of our best performing model were tested in a benchmark session against the tool ModPred (Pejaver *et al.*, 2014), the only ADP-ribosylation site predictor available in the literature. The capacity of both models to correctly predict the experimental data from the Yu, Nielsen and Kraus datasets was assessed by comparing ROC curves.

## 4 Implementation

Development of the ADPredict tool was performed in workflow programming using the BIOVIA Pipeline Pilot program. All calculations were run on a Dell PowerEdge r820 server, equipped with a 2.40 GHz 32-thread Xeon E5-4640 processor and 512 GB of RAM in a Windows server 2012 environment. The Mks SIMCA program was used for PCA model analysis and evaluation (Eriksson *et al.*, 2006). MOE and Schrödinger program suites were used for visual inspection of 3-D structures, automated protein fragment extraction, and property calculations. The ADPredict website (www.ADPredict.net) is implemented using LAMP (Linux Apache MySQL PHP), an open source Web development platform that allows a fluent and responsive user experience in displaying and handling the output data, which in this case are calculated on the fly in a completely automated Pipeline Pilot workflow. It runs on an Apache/2.4.6 (CentOS) PHP/5.4.16 Server.

## 5 Results and discussion

### 5.1 Primary sequence-related models

**Fig. 3.**
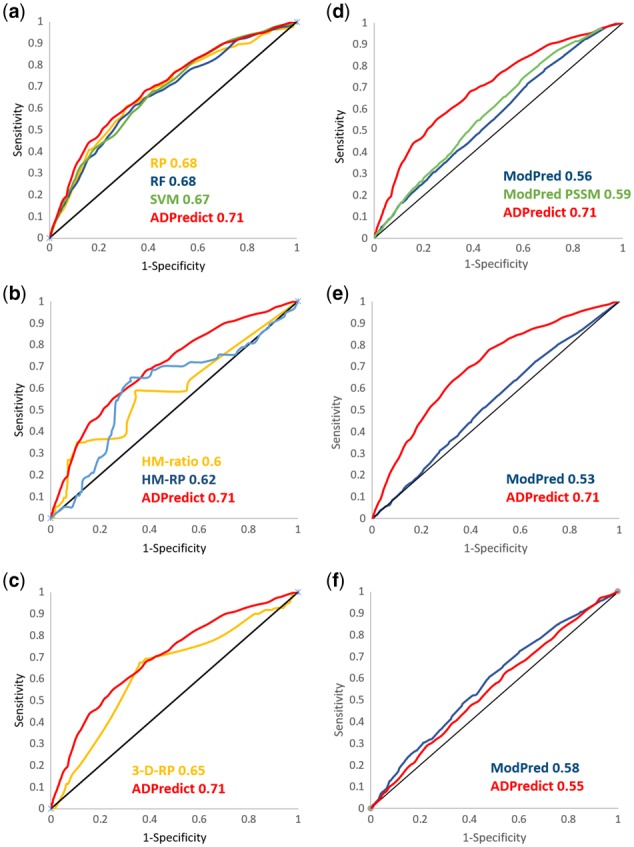
**Cross-validation (a)–(c) and Benchmark (d)–(f) ROC curves. (**a) Primary sequence-based models, (b) secondary structure-based models and (c) 3-D based model L1O results. Comparative analysis of the ADPredict and, ModPred performances in predicting (d) Yu, (e) Kraus and (f) Nielsen datasets. ModPred PSSM performance is evaluated for the Yu dataset only

For the developed models based on amino acid physicochemical properties, the three machine-learning techniques showed comparable global performances, according to the ROC values, whereas they had quite different EF values, indicating dissimilar performance in retrieving TPs within the top three ranked position. The mean ROC scores were very similar and robust (Fig. 3a), indicating that the overall prediction capacity was well performing and reliable (0.68, 0.8 and 0.67 for RP, RF and SVM, respectively). However, the RP model in particular showed a higher percentage of correctly predicted proteins—50.6% compared to 31 and 26.8% identified by RF and SVM (Table 2). This behavior can be interpreted by considering the different EF and relative SDEV values. The EF values of the RF and SVM models (2.78 and 3.10, respectively) were within the very high- and low-scored predictions, as shown by their high SDEV values (7.05 and 8.16, respectively), reflecting the capacity to correctly predict a lower number of proteins but with a high recall of sites compared to the RP model. This means that RF and, to a greater extent, SVM are more elitist models than RP, and when they correctly predict a site they do it with high confidence at the top ranked positions. On the other hand, the RP model had a more constant prediction performance, with an EF of 2.40 and a relative SDEV of 2.60 (Table 2). All three methods were also checked for their robustness in the bootstrapping stage. For all the methods, the EF, ROC and the relative SDEV-all were similar to those of the L1O session. A low SDEV-mean—especially for the ROC—supports how the performance of the models does not depend on the data of sampling (Table 2). For all the models, the VLS 9 represented the best-performing string length. In light these results, performing with comparable efficacy but still showing different recall, all three methods advanced to the next level of the study.

**Table 2.** Cross-validation results

	EF (TOP3)					Proteins with EF > 2 (%)	ROC				
	L1O		L10%O				L1O		L10%O		
	Mean	SDEV	Mean	SDEV(Mean)	SDEV(All)		Mean	SDEV	Mean	SDEV(Mean)	SDEV(All)
AAD-RP	2.406	2.601	2.453	0.536	3.041	50.6	0.684	0.230	0.666	0.042	0.236
AAD-RF	2.778	7.046	2.499	1.307	7.481	31.0	0.684	0.224	0.671	0.039	0.238
AAD-SVM	3.104	8.157	2.884	1.432	8.733	26.8	0.671	0.247	0.666	0.041	0.250
HM-ratio	2.392	4.245	2.393	1.846	3.949	32.4	0.603	0.281	0.613	0.126	0.283
HM-RP	1.707	1.324	1.643	0.590	1.343	37.8	0.622	0.214	0.607	0.093	0.214
3-D-RP	1.745	1.498	1.918	0.919	2.068	48.1	0.650	0.233	0.654	0.105	0.231
ADPredict	3.427	7.331	3.395	1.445	8.206	33.5	0.707	0.234	0.7	0.04	0.235

*Note:* Resuming table of the model performance in predicting ADP-ribosylated sites of the training set, in both a L1O and a L10%O cross-validation sessions. Selected models for each class of properties are reported, as well as the consumptive model, ADPredict. EF and ROC values, along with relative SDEV values, are calculated as evaluation functions. Proteins with an EF higher than two are considered correctly predicted and are here reported as percentage of the training set.

### 5.2 Secondary structure-related models

Initially being a simple value to calculate, the HM-ratio was applied as a single-level binary classification, with the threshold set to 1, consistent with the intrinsic meaning of the descriptor. Also in this case, proteins were considered correctly predicted when characterized by an EF value greater than two but selecting the top ranked residues only if their HM-ratio value was greater than one (meaning an imbalance in HM frequencies in favor of TPs). Only slightly different from the primary sequence-based models, the best-performing length for the secondary structure-based algorithms was VLS 11. Among several explored approaches, the best strategy was the one segmenting the 11-residue sub-sequences into three regions (three, five and three residues long, respectively), finally coded by a three-letter hashed motif, that, considering the full set of permutations with repetition of 4-fold (H, E, T and C) and the uncertainty (0), amount to a total of 125 possible fingerprints. At the feature-extraction level, HM-ratios were calculated as the ratio between TP and TN hashed motif frequencies. The resulting values allowed two kinds of information to be collected: (i) the folds with the highest frequencies among TPs can be noted as the most eligible ‘shapes’ for ADP-ribosylation and (ii) the higher the HM-ratio for a given fold, the more this discriminates for a modified residue, thus allowing to design a predictive model. The frequency among TNs cannot be calculated directly because TNs are in speculative annotations attributed to the unmodified residues, which in turn can be unmodified for many different reasons (for example, the accessibility to the enzyme). Analyzing the HM distribution across TPs in the training set, 18% of them were represented by only one site. Thus, to properly perform the L1O evaluation, the proteins containing these singletons were excluded from the set to avoid the case in which the test set is out of the model’s applicability domain. The resulting model showed a ROC mean value of 0.60 with a SDEV of 0.28 and an EF value of 2.39 with a SDEV of 4.24, correctly predicting 32.4% of the proteins (Fig. 3b;Table 2). Later, a classification RP tree method was exploited to avoid imposing a specific HM-ratio threshold. Here, multiple values were allowed, as selected by the model at each branching node, and predictions were expressed as probabilities from 0 to 1, allowing a performance comparison with the other models.

The resulting model consisted of a tree with four branches, identifying two main HM-ratio ranges in which an enrichment of TPs with respect to random incidence was observed, consistent with the meaning of the descriptor: (i) values higher than 1.96 (Supplementary Fig. S1, box #6) and (ii) values between 1.11 and 1.96 (Supplementary Fig. S1, box #10). At the same time, a TN enrichment was detected for values lower than one (Supplementary Fig. S1, boxes #1 and #9). This model showed a mean ROC of 0.62 (SDEV of 0.21) and an EF of 1.71 (SDEV of 1.32), correctly predicting 37.8% of the proteins (Fig. 3b). The L10%O calculation supported the reliability of the models, with ROC and EF values of 0.61 and 1.64 and a SDEV-mean of 0.09 and 0.59, respectively (Table 2). Thus, the VLS 11 HM-RP model was selected as the best-preforming model for secondary structure-related information. In light of these results, the proposed hashing approach resulted to properly catch the secondary structure information of the considered subsequence and allowed to generate a reliable descriptor, HM-ratio, exploitable in discriminating between putative and not modifiable residues.

### 5.3 3-D descriptor related models

3-D property-based algorithms were developed in a manner similar to the physicochemical property session using three different machine learning techniques (RF and SVM results are not shown). Out of the three techniques applied, the RP method resulted in the best-performing model. At the L1O level, it showed a mean ROC value of 0.65 and a mean EF value of 1.58 (SDEV of 1.68) and resulted in the correct prediction of 37% of the proteins in the set (Fig. 3c). Also in this case, the L10%O calculation supported the robustness of the model, with a ROC of 0.65, an EF of 1.92 and a SDEV-mean of 0.1 and 0.92 (for ROC and EF, respectively) (Table 2). On these results, the use of 3-D descriptors as well was proved to be meaningful and the resulting model was promoted to the following stages of the study.

### 5.4 External predictions

To further prove their predictive capacity, the five best-performing models (the three AAD based, the HM-ratio-based RP and the 3-D-RP) were tested in predicting the external datasets of Kraus and Nielsen. Of note, being these two datasets generated with different experimental strategies respect to those of the training set, using the predictive models offered also the chance to estimate if and how much ADP-ribosylation property patterns really diverge along with the experimental conditions Again, the EF and ROC functions were calculated to evaluate the predictive performance.

#### 5.4.1  AAD-based models

The three AAD-based predictive models were used for the prediction of both Kraus and Nielsen dataset, obtaining encouraging results (Table 3). In more detail, the RP, RF and SVM models correctly predicted 51.8, 30 and 26.4% of the proteins in the Kraus set, compared with 25.1, 10.7 and 11.5% of those in the Nielsen list, respectively. Consistent with the local predictions, global measures were also better when predicting proteins in the Kraus set, as indicated by ROC values ranging from 0.66 for the SVM model to 0.70 for the RP model. The best model in predicting Nielsen was still RP, but this model only had ROC values of 0.54 (Table 3). These results, especially in the case of Kraus dataset, further confirmed the predictive capacity of AAD-based models, successfully retrieving the effective modified sites of an external set as numerous as the training one.

**Table 3.** External validation results

	Nielsen dataset					Kraus dataset				
	EF (TOP3)		Proteins with EF > 2 (%)	ROC		EF (TOP3)		Proteins with EF > 2 (%)	ROC	
	Mean	SDEV		Mean	SDEV	Mean	SDEV		Mean	SDEV
AAD-RP	1.190	2.018	25.1	0.538	0.243	2.569	2.617	51.8	0.696	0.244
AAD-RF	1.289	6.998	10.7	0.532	0.249	3.176	6.823	30.0	0.689	0.248
AAD-SVM	1.387	7.013	11.5	0.534	0.262	2.585	5.991	26.4	0.661	0.249
HM-ratio	1.532	2.897	26.3	0.545	0.316	1.037	2.293	16.8	0.510	0.269
HM-RP	1.413	2.604	23.7	0.549	0.304	1.033	1.698	19.3	0.500	0.250
3-D-RP	0.994	1.572	19.5	0.529	0.261	1.987	2.150	46.2	0.656	0.255
ADPredict	1.399	6.987	12.1	0.547	0.247	2.624	5.628	28.3	0.706	0.228

*Note:* Resuming table of the model performance in predicting ADP-ribosylated sites of the two external datasets. EF and Roc values, along with relative SDEV values, refers to a L1O session.

#### 5.4.2 Secondary structure-based models

Similar to what was done for the Yu set, both TP-specific and the TP/TN discriminating folds were inspected in the Kraus and Nielsen sets. The Pearson correlation coefficient, as computed for each pair of sets, showed a quite strong positive correlation of the HMs for TP, whereas the correlation values dropped when considering the HM-ratio. This can be probably explained considering the paucity of overlap of modified sites among the three sets and the intrinsic spread distribution of the descriptor itself. This is more evident focusing the analysis on the 43 proteins commonly modified in all the studies. In contrast, HMs among TNs appeared to be highly correlated and the analysis of the entire human proteome, checked in the PDB databank, allowed us to further confirm the observed global distribution (see Supplementary Table S3 for more details). The Kraus and Nielsen datasets were then used as external test sets to check the prediction capacity of secondary structure-based models. In order not to bias the results by predicting an object that had been used in the training set, proteins in common with the Yu list were subtracted from the Kraus and Nielsen sets. The HM-RP model showed similar and poor results in predicting Kraus and Nielsen unique proteins, as evidenced by ROC values of 0.5 and 0.55, respectively. From the local model point of view, the gap between the trends grows, with an EF value of 1.41 for Nielsen and 1.03 for Kraus. The model had better performance in predicting the Nielsen than the Kraus set, being successful in 23.7% of the cases compared to 19.3% (Table 3).

These results, in line with what observed in the correlative analysis of the folds frequency across the different datasets, showed HM-ratio failing in predicting the external sets. However, it has to be considered the intrinsic spread distribution of the HM descriptors (125 different elements in which to catalog, with often an even lower number of samples), limit that we are confident will be overcome as the training set grows.

#### 5.4.3 3-D descriptor-based models

Among the three machine learning techniques applied for developing the 3-D properties based models, the RP was selected as the best performing in predicting Yu data (L1O). Thus, only RP was exploited for the external predictions of Kraus and Nielsen datasets. As showed by the resulting ROC and EF values, once again, the model performance was better in predicting the Kraus set (ROC of 0.66, EF of 1.99, correctly predicting 46.2% of proteins) than the Nielsen set (ROC of 0.53, EF of 1, correctly predicting 19.5% of proteins) (Table 3). Once again, similarly to what highlighted for the physicochemical properties by the AAD-based predictive models, 3-D-RP as well showed how the three-dimensional properties of Yu and Kraus datasets appear to be more aligned then those of Yu and Nielsen, in line with the respective trend in TPs overlap among datasets.

### 5.5 *Consensus* model, ADPredict

To combine the contribution of each selected model, a *consensus* model, from here on named ADPredict, accounting for the mean prediction of all models (namely the three AAD-based, the RP developed on HM-ratio and the 3-D-RP), was generated and tested. In predicting the Yu dataset, the ADPredict model outperformed all the other models in terms of global performance, showing a mean ROC value for the L1O stage of 0.71 (Fig. 3; Table 2). In line to what observed for each single model, the L10%O of *consensus* model as well proved it to be robust and to perform better of each individual predicting model, as reported by both ROC and EF higher values and SDs in line with previously described ones (Table 2).

We identified 0.4 as the significance threshold for the prediction, the value that corresponds to an EF value larger than two. The 0.4 threshold maximize the number of correctly predicted modified sites while minimizing the number of FNs. For this value, sensitivity (or true positive rate—TPR), specificity (or true negative rate—TNR) and accuracy showed values of 0.46, 0.78 and 0.77 respectively. Thus, sites with an ADPredict score equal to or higher than 0.4 are putative ADP-ribosylated sites. ADPredict model was mostly driven by the AAD models because only a minor portion of the set had resolved structures. Its prediction capacity was better than the RF and SVM models, with an improved EF value (3.43, SDEV of 7.3), and it correctly predicted 33.5% of the proteins (Table 3). ADPredict model was then tested with the two external datasets, resulting to perform better in predicting the Kraus dataset than for the Nielsen dataset, with ROC values of 0.71 and 0.55, EF values of 2.6 and 1.4, and correctly predicting 28.3 and 12.1% of the proteins, respectively (Table 3). On these grounds, we definitively confirmed that the Yu and Kraus ADP-ribosylation profiles are more similar to each other than to the Nielsen one. In light of these results, ADPredict model represents the best predictive metrics for ADP-ribosylation site prediction. It offers a clear picture of the ADP-ribosylation profile of the target of interest, at the same time accounting for all the diverse features considered in this study. It is the main output of the online application.

### 5.6 Benchmark results

To determine how well ADPredict performs compared to only other available ADP-ribosylation prediction tool, ModPred (Pejaver *et al.*, 2014), a benchmark session was carried out. The Yu, Kraus and Nielsen datasets were predicted using with the ModPred tool and the results were compared with those from our final model (Fig. 3d–f). ModPred resulted in ROC values of 0.56 and 0.53 for the Yu and Kraus datasets, respectively, compared to 0.71 and 0.70 obtained with ADPredict (Fig. 3d and e). In contrast, both models obtained comparably poor results for the Nielsen dataset, as shown by ROC values of 0.58 for ModPred and 0.55 for ADPredict (Fig. 3f). Furthermore, the ModPred-PSSM model, which was reported to have higher performance by Pejaver and colleagues, was used to predict the Yu dataset and only slightly improved the result, yielding a ROC value of 0.59 (Fig. 3d), which was still markedly lower than the ADPredict value of 0.71.

## 6 Web server tool implementation

**Fig. 4.**
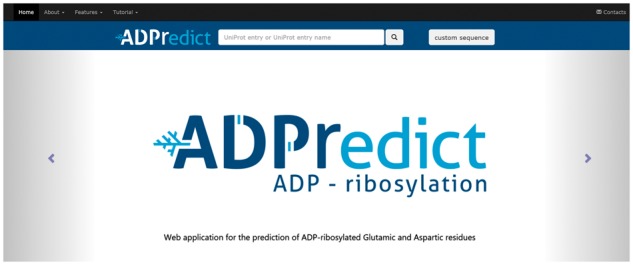
ADPredict web application homepage semi-screenshot

ADPredict is available as a web application, freely accessible at ADPredict.net (Fig. 4). The user can perform the search by entering the UniProt entry or the UniProt entry name of the protein of interest and entering a custom Fasta sequence. In this second case, only standard amino acids are allowed and only AAD-based predictive models will be calculated (Supplementary Fig. S3a). The human 60S ribosomal protein L11 (rl11_human, P62913), included in the Kraus dataset, is reported here as an example of an ADPredict output (Supplementary Fig. S3b–d). Three residues are predicted to be modified and the first two of these are experimentally validated targets of ADP-ribosylation (Supplementary Fig. S4). Initially, ADPredict reports all glutamic acid and aspartic acid residues present in the protein primary sequence and the related information. In more detail, the ADP-ribosylatable residues are listed in a table that reports, for each site (i) the position within the sequence, (ii) the VLS in which it is located (VLS 11 is preferred, otherwise VLS 9, if lower the site is marked as discarded and not predicted), (iii) the relative secondary structure string (if available) and, in that case, (iv) the selected resolved structure (PDB ID) (Supplementary Fig. S3b). In addition to the table, a pie chart summarizes the count of residues of interest, the information they come with and the models available for the calculation. Submitting the query leads to the prediction output, represented by both a plot and a table, which are completely interactive and available for download (Supplementary Fig. S3c and d). The web server by default will calculate the ADPredict model and the other five models described earlier. An easy user guide can be found on the website in the tutorial section.

## 7 Conclusions

In this study, we exploited multiple machine learning techniques to develop the ADPredict, a tool for the prediction of ADP-ribosylatable aspartic acid and glutamic acid residues in a target protein. ADPredict relies on physicochemical properties, combining them with structure-related information when available. Extensive bootstrapping and external predictions support the effectiveness of its predictive power, and the benchmarking results indicate it is the best tool in the field.

ADPredict applies at different levels of molecular and cellular biology. At a more comprehensive stage, when studying a specific pathway known to be regulated by ADP-ribosylation, it helps focusing the attention on the more promising substrate(s) among several putative players, so supporting the early investigation stages of cellular pathways. Besides, at a more specific level, such as the study of a known ADP-ribosylation target, the tool allows identifying this modification event at a residue level, a step needed to describe the molecular mechanisms underlying the pathways of interest. In addition, the high selectivity of the tool in identifying sites more prone to be modified strongly reduces the likelihood of false positive results, thus facilitating the validation of the prediction through mutagenesis analysis and so allowing to study the event of interest without altering the environmental steady conditions.

Of note, the comparison of the ADP-ribosylation datasets indicates high variability, depending on the biochemical environment (cell type, physio-pathological conditions) and the experimental procedures used for enrichment and characterization of the ADP-ribosylated sites. Indeed, both qualitative and quantitative experimental evidence of this variability has recently been published (Bilan *et al.*, 2017). However, despite this variability, we observed that an ADP-ribosylatable site possesses a basal set of physicochemical properties as well as an opportune shape and 3-D features that, independent from the experimental conditions, predispose it to be modified. Aiming to improve the predictive performance of our model, the property space on which it was developed will be enlarged by including the novel information contributed by the Kraus and Nielsen datasets, as identified by the external prediction results. We are confident that a richer training set would allow to increase the power of our prediction tool and, particularly, it would offer the chance to overcome the observed limitation of the well promising in-house generated structure based descriptor, HM-ratio. At the same time, the experimental strategy used by Nielsen offers the chance to explore the ADP-ribosylation trend in lysine and arginine residues. Therefore, we intend to extend the ADPredict applicability to basic amino acids and emerging new ADP-ribosylated residues such as serine (Bilan *et al.*, 2017b; Bonfiglio *et al.*, 2017) to fully support biologists of the field in the study of this molecular event.

## Supplementary Material

Supplementary Data
