# Supplementary material for: ADPredict: ADP-ribosylation site prediction based on physicochemical and structural descriptors
Source: Bioinformatics. 2018 Mar 15;34(15):2566–74. doi: 10.1093/bioinformatics/bty159 (PMC6061869; doi:10.1093/bioinformatics/bty159)
Supplement: Supplementary Data [file bty159_supp.zip › bty159-suppl_data/ADPredict Supplementary.doc]

# *ADPredict*: ADP-ribosylation site prediction based on physicochemical and structural descriptors

Matteo Lo Monte^1,†,*^, Candida Manelfi^2,†^ , Marica Gemei^2,3^, Daniela Corda^1^ and Andrea Rosario Beccari^1,2^

^1^ Institute of Protein Biochemistry, National Research Council, Naples, Via P. Castellino 111, 80131, Italy

^2^ Dompé Farmaceutici SpA, L'Aquila, Via Campo di Pile, 67100, Italy

^3^ Università degli Studi di Milano, Dipartimento di Scienze Farmaceutiche, Via Mangiagalli 25, 20122, Milano

*To whom correspondence should be addressed.

^†^ The authors wish it to be known that, in their opinion, the first two authors should be regarded as Joint First Authors.

**Supplementary data**

**Table S1**. Secondary structure coding scheme

**Table S2**. 3-D properties calculated as 3-D-descriptors

**Table S3.** Linear correlation analysis

**Table S4.** Protein datasets

**Figure S1.** Schematic diagram of the HM-RP tree

**Figure S2.** Dataset comparative analysis

**Figure S3.** ADPredict webserver screenshots

**Figure S4.** 3-D representation of the rl11_human crystal structure

**Table S1. Secondary structure coding scheme**

| 2^ndary^ structure | PDB  Symbol | Simplified  Symbol | Simplified  Fold |
| --- | --- | --- | --- |
| α-Helix | H | H | Helix |
| 3_10_Helix | G | H | Helix |
| π-Helix | I | H | Helix |
| Turn | T | T | Turn |
| Bend | S | T | Turn |
| Beta Bridge | B | E | Strand |
| Extended Strand | E | E | Strand |
| - | - | C | Coil |

The canonical secondary structure classification based on seven motifs is translated into a simpler four-motif classification, collapsing similar classes such as the different kinds of helices (α-helix, 310 helix and π-helix ), turns (H-bonded or non H-bonded turns) and strands (Beta bridge or extended strand) into more general Helix, Turn and Strand categories.

**Table S2.** **List of the 3-D parameters calculated as 3-D-descriptors**

| Program | Scoring function |
| --- | --- |
| MOE | Free ASA* |
|  | GxG ASA |
|  | Exp ASA |
|  | % Exposure |
|  | % Buried |
|  | Interactions |
| Residue Analysis Panel (Schrödinger Biologics) | Hydropathy |
|  | Potential Energy |
|  | Internal Energy |
|  | Interaction Energy |
|  | Atomic SASA** nonpolar |
|  | Atomic SASA polar |
|  | SASA total |
|  | Rotatable bonds |

*ASA: Accessible Surface Area

**SASA:Solvent Accessible Surface Area

**Table S3**. **Linear correlation analysis (Pearson coefficient)**

**S3, a.** Linear correlation of Hashed Motif frequencies in Yu, Kraus and Nielsen’s TPs

|  | HM frequency in Yu’s TP | HM frequency in Kraus’s TP | HM frequency in Nielsen’s TP |
| --- | --- | --- | --- |
| HM frequency in Yu’s TP | 1 | 0.69818 | 0.6049 |
| HM frequency in Kraus’s TP | 0.69818 | 1 | 0.64251 |
| HM frequency in Nielsen’s TP | 0.6049 | 0.64251 | 1 |

**S3, b.** Linear correlation of Hashed Motif frequencies in Yu, Kraus and Nielsen’s HM-ratio

|  | HM frequency in Yu’s HM-ratio | HM frequency in Kraus’s HM-ratio | HM frequency in Nielsen’s HM-ratio |
| --- | --- | --- | --- |
| HM frequency in Yu’s HM-ratio | 1 | -0.028362 | 0.066187 |
| HM frequency in Kraus’s HM-ratio | -0.028362 | 1 | -0.066393 |
| HM frequency in Nielsen’s HM-ratio | 0.066187 | -0.066393 | 1 |

**S3, c.** Linear correlation of Hashed Motif frequencies in Yu, Kraus and Nielsen’s TNs

|  | HM frequency in Yu’s TN | HM frequency in Kraus’s TN | HM frequency in Nielsen’s TN |
| --- | --- | --- | --- |
| HM frequency in Yu’s TN | 1 | 0.99087 | 0.98895 |
| HM frequency in Kraus’s TN | 0.99087 | 1 | 0.99553 |
| HM frequency in Nielsen’s TN | 0.98895 | 0.99553 | 1 |

**S3, d.** Linear correlation of Hashed Motif frequencies in Yu, Kraus and Nielsen’s TPs, accounting for in common modified proteins only.

|  | HM frequency in Yu’s TP | HM frequency in Kraus’s TP | HM frequency in Nielsen’s TP |
| --- | --- | --- | --- |
| HM frequency in Yu’s TP | 1 | 0.62289 | 0.47724 |
| HM frequency in Kraus’s TP | 0.62289 | 1 | 0.54133 |
| HM frequency in Nielsen’s TP | 0.47724 | 0.54133 | 1 |

*p*-values are always lower than 0.01.

Despite the relative frequencies, which strictly depend on the diverse nature of the samples, it is of note that the most represented hashed motifs in TPs are much the same among the datasets. Only seven motifs, in fact, can be found in the top five most populated sites of all samples, supporting the existence of preferred profiles among the of ADP-ribosyltransferase targets (CHH, THH, CCC, HHT, HHH, HHC and TTC).

The analysis of a database of 152,603 Glu/Asp-centered sub-sequence, retrieved form PDB databank and annotated for the protein folds, allowed us to further confirm the observed global distribution. Yu, Kraus, and Nielsen TNs, in fact, appeared to be perfectly aligned to the HM trend in the human resolved proteome, as clearly supported by very high linear correlation values (approximately 0.98, data not shown). These observations suggest that there is no bias related to the nature of samples.

**Table S4**. **Protein datasets**

Refined training set (sheet 1) and both external validation datasets (Kraus and Nielsen, sheet 2 and 3, respectively) are available in xls format. For each protein, gene name and Uniprot entry are listed, along with the modified sites and relative position within the sequence, the primary sequence and the sequence length.

See Protein Datasets.xls in Supplementary material.

**Figure S1. Schematic diagram of the HM-RP tree**

Result of the RP model computed on VLS 11. Rounded, green boxes indicate a range window in which there is an improvement in TP/TN imbalance; red boxes indicate the opposite situation.

**Figure S2. Dataset comparative analysis**

Venn diagrams of the ADP-ribosylated protein (a and c) and ADP-ribosylated site (b and d) overlap among the three sets resulting from the pre-processing stages, considering both the entire datasets (a and b) and the structure resolved datasets (c and d).

The study published by Kraus and colleagues (Gibson et al., 2016) describes an in vitro mass spectrometric approach to investigate the nuclear ADP-ribosylome pattern of HeLa cells in a PARP-selective manner using engineered NAD+ analog-sensitive PARPs (PARP1-3). On the other hand, the study published by Nielsen and colleagues (Martello et al., 2016) reports an experimental workflow for the analysis of endogenous ADP-ribosylation in human cells under oxidative stress conditions and in mouse liver tissues. For what concerns the first one, once we pre-processed the published dataset, preparing them for our analysis –using the same protocol applied in refining the Yu dataset–, a total of 1,150 refined ADP-ribosylated sites, distributed across 465 proteins, resulted (accounting for both aspartic acid and glutamic acid residues). In parallel, the refined list of the second dataset resulted into 1,137 ADP-ribosylated sites, distributed across 461 proteins. Importantly, when contiguous modifiable residues within an identified modified peptide were not precisely resolved due to resolution limits of the exploited analytical strategies, each site was considered as TP and treated as independent objects in developing the models.

Once pre-processed, the Yu set accounted for 317 proteins, whereas the Nielsen and Kraus sets involved 461 and 465 proteins, respectively. The Yu and Nielsen sets appeared to have 107 proteins in common, but with a marked difference in terms of modified sites: in the Yu list, these 107 proteins accounted for a total of 520 ADP-ribosylation sites, whereas the same proteins comprised 358 modified sites in the Nielsen set. Moreover, only 71 sites were the same in the two ensembles, so only 34 of 107 proteins shared at least one site. The Yu set shared 84 proteins with the Kraus list, of which 43 had at least one modified site in common, for a total of 77 sites in common. Completing the pairwise analysis, the Kraus and Nielsen sets shared 114 proteins, with 40 having at least one site in common. Considering all three sets together, the number of proteins in common drops to 43, with only seven having at least one similar modified amino acid, for a total of 12 shared TPs.

**Figure S3, a-d. ADPredict webserver output**

Rl11_human ADP-ribosylated site prediction as output, step by step, by ADPredict web server. a) A semi-screenshot showing the custom query page of the ADPredict webserver. b) List of the acidic residues in the protein of interest, annotated for their sub-sequence length, secondary structure, and 3-D information availability. c) ADP-ribosylated site prediction table (DATA tag). d) ADP-ribosylated site prediction chart (PLOT tag); here ADPredict model results are reported (gray bars) along with the results using the other models (colored lines). Residues with an ADPredict score above 0.4 are highlighted by coloring the bar, graded from cyan to blue with increasing score.

**a)**

**b)**

**c)**

**d)**

**Figure S4.** **3-D representation of the rl11_human crystal structure**

Ribbon cartoon of the rl11 crystal structure (PDB ID: 4XXB). The VLS 11 string, centered on the key residues, is reported in secondary structure color-coded ribbon: helix in red, coil in blue, and strand in yellow. The three predicted ADP-ribosylation sites are highlighted: Glu28 and Glu41, which were the second and first in the prediction ranking, respectively, and correspond to the two modified residues of the protein, are colored in blue; Glu80, a false positive prediction, is colored in cyan.
